# Angiotensin-(1–7) and angiotensin II induce the transdifferentiation of human endometrial epithelial cells *in vitro*

**DOI:** 10.3892/mmr.2014.2128

**Published:** 2014-04-09

**Authors:** TIEYING SHAN, LEI ZHANG, CHUNFANG ZHAO, WEI CHEN, YANAN ZHANG, GUIYING LI

**Affiliations:** 1 Department of Histology and Embryology, Hebei Medical University, Shijiazhuang, Hebei 050017, P.R. China; 2 Department of Histology and Embryology, Hebei Engineering University, Handan, Hebei 056002, P.R. China

**Keywords:** angiotensin-(1-7), angiotensin II, human endometrial epithelial cells, transdifferentiation, E-cadherin, fibronectin, collagen type I, α-smooth muscle actin

## Abstract

Intrauterine adhesions (IUA) may be caused by endometrial stromal cell proliferation, increases in myofibroblasts or increases in extracellular matrix secretion. However, the specific mechanisms underlying the development of IUA have yet to be elucidated. The present study identified that angiotensin *(*Ang) II is capable of promoting endometrial epithelium cell (EEC) proliferation and the transdifferentiation of EECs into myofibroblasts. Furthermore, the present study found that Ang II increased the expression of the myofibroblast specific protein α-smooth muscle actin (α-SMA), decreased the expression and secretion of E-cadherin, and increased the synthesis of collagen type I (Col I) and fibronectin (FN). However, Ang-(1–7) was observed to inhibit Ang II-induced proliferation and transdifferentiation of EECs, decrease the expression of α-SMA, increase the expression of E-cadherin and decrease the synthesis and secretion of Col I and FN. These findings suggest that Ang-(1–7) is capable of inhibiting the Ang II-induced proliferation and transdifferentiation of human EECs and decreases in Col I and FN secretion. The present study may provide insight into the mechanism underlying endometrial fibrosis.

## Introduction

Intrauterine adhesions (IUA) have various causes including, incorrect uterine cavity surgery, induced abortion and secondary infections. Patients with IUA often exhibit reduced menstruation, amenorrhea and infertility. The disease was first described by Asherman in 1948 and is clinically referred to as Asherman’s syndrome (1). Histologically, Asherman’s syndrome is a condition in which the endometrium becomes fibrosed (2), with the endometrial stroma becoming largely replaced by fibrous tissue (3). Under hysteroscopy, patients with Asherman’s syndrome exhibit pale scar tissue distributed in islands among the normal endometrium, with severe IUAs forming thick bands (4). At present, the mechanism underlying intrauterine fibrosis is unclear and there is a lack of effective means for the diagnosis and prevention of the disease worldwide. Therefore, basic and clinical research is required in order to elucidate the mechanism underlying endometrial fibrosis and develop novel preventative and therapeutic strategies.

Ang II is a key member of the renin-angiotensin-aldosterone system (RAAS) and has roles in several processes, including vasoconstriction, cell proliferation, fibrinolysis and blood pressure elevation. Ang II has been reported to be involved in the fibrosis of certain organs, including the heart (5), kidney (6,7) and liver (8,9); therefore, Ang II has an important role in the process of organ fibrosis. Ang II has not only been reported to promote the proliferation of various mesenchymal cells, but also to cause the accumulation of extracellular matrix components, leading to fibrosis (10–12). Ang-(1–7) is a novel member of the RAAS and has been shown to be an endogenous antagonist of Ang II. Ang-(1–7) has been reported to have an important physiological role in dilating blood vessels, lowering blood pressure, inhibiting vascular smooth muscle cell and cardiac fibroblast proliferation (13), inhibiting myocardial cell hypertrophy and reducing ventricular remodeling (14,15). Ang II has increasingly been reported to have an important role in the development of tissue fibrosis and the physiological antagonism of Ang II by Ang-(1–7) *in vivo* may be important for inhibiting tissue fibrosis.

The present study aimed to investigate the effect of Ang-(1–7) and Ang II on human EECs in order to develop a theoretical basis for the prevention and treatment of endometrial adhesion.

## Materials and methods

### Endometrial specimen collection

Endometrial tissues were obtained by hysterectomy from 20 females with uterine fibroids at The Second Affiliated Hospital of Hebei Medical University (Shijiazhuang, China) between September and December 2012. This study was approved by the Ethics Committee of Hebei Medical University and informed consent was obtained from all patients prior to hysterectomy. The patients with uterine fibroids were aged between 30 and 45 years old with menstrual cycle durations between 24 and 35 days (mean, 28 days). None of the patients had received any hormonal treatment for at least three months prior to the hysterectomies. Postoperative pathological analyses were performed to confirm that the endometrial tissues exhibited hyperplasia and were disease-free. Endometrial tissue samples were collected under aseptic conditions from the uterus and immediately placed in Dulbecco’s modified Eagle’s medium/Nutrient Mixture F-12 (DMEM/F12; Gibco-BRL, Grand Island, NY, USA) containing 10% fetal calf serum, penicillin and streptomycin (100 mg/ml; Gibco-BRL) in an ice bath. Samples were transported to the laboratory within 2 h.

### EEC isolation, purification and culture

Following several washes with phosphate-buffered saline (PBS), tissues were cut into 1–2-mm^3^ pieces using sterile scissors and incubated in 5 ml DMEM/F12 containing 0.2% collagenase I (Sigma-Aldrich, St. Louis, MO, USA) in an incubator with shaking for 60 min at 37°C with 5% CO_2_. Throughout the incubation process, the tissue pieces were pipetted gently to disperse the cells. The whole cell suspension, which contained EECs and endometrial stromal cells (ESCs), was centrifuged at 500 × g for 5 min. The supernatant containing the ESCs was discarded, while the precipitate was resuspended in a culture flask with 3 ml DMEM/F12 containing 10% fetal bovine serum (FBS; Gibco-BRL) and 1% penicillin and streptomycin. The EECs attached to the culture flask were washed several times with serum-free DMEM/F12 to remove the red blood cells.

The Trypan blue exclusion assay (Zhongshan Biotech Co., Ltd., Beijing, China) was performed to assess the proportion of the active cells. A small number of the cells were then seeded onto 6-well plates containing coverslips and incubated in an atmosphere of 5% CO_2_ at 37°C for cell type characterization and purity analyses.

### Morphological observation of EECs

EECs were cultured in the aformentioned medium for 0 and 5 days and were stained with hematoxylin and eosin (H&E). Endometrial cell morphology and structure were observed using an inverted phase-contrast microscope (Nikon, Tokyo, Japan) and a light microscope (Olympus, Tokyo, Japan).

### Identification of EECs

To identify EECs and assess their purity, immunocytochemical staining was performed. PBS was used as a negative control instead of primary antibodies. Cells that had been cultured on the cover slides were fixed using 4% paraformaldehyde and treated with 0.25% Triton X-100. Subsequent to blocking using 5% normal goat serum for 20 min at 37°C, cells were incubated with rabbit rabbit anti-human cytokeratin (dilution, 1:100) and vimentin (dilution, 1:100) primary antibodies (Zhongshan Biotech Co., Ltd.) at 4°C overnight. Cells were then incubated with goat anti-rabbit immunoglobulin G (IgG; dilution, 1:100; Boster Biological Technology Co., Ltd., Wuhan, China) for 20 min at 37°C and stained with 3,3′-Diaminobenzidine (DAB; 5 mg/ml; Sigma-Aldrich) for 5 min at room temperature. The specimens then underwent three 5-min washes with PBS and were observed using light microscopy.

### Experimental groups

EECs were divided into four groups according to the different treatment interventions. The groups and treatment conditions were as follows: Control group, treated with serum-free DMEM/F12; the Ang II group, treated with Ang II and serum-free DMEM/F12; the Ang-(1–7) group, treated with Ang-(1–7) and serum-free DMEM/F12; and Ang II + Ang-(1–7) group, treated with Ang II, Ang-(1–7) and serum-free DMEM/F12. The final concentrations of Ang-(1–7) and Ang II were 10^−5^ and 10^−6^ mol/l, respectively.

### Cell proliferation assay

EECs (4×10^4^/ml) were seeded on 96-well plates and cultured in serum-free DMEM/F12 for 24 h, in order to synchronize their growth. The EECs were divided into the aformentioned four groups, with six wells/group and cultured for 24, 48 and 72 h. MTT (5 mg/ml) was then added to each well and the plates were incubated at 37°C for 2 h. The medium was subsequently replaced with 150 μl dimethylsulfoxide and agitated for 10 min. The absorbance at 560 nm was measured using a microplate reader (Packard Instrument Co., Inc., Meriden, CT, USA).

### Immunocytochemistry

The four groups of cells were cultured for 72 h. PBS was used as a negative control instead of primary antibodies. Cells that had been cultured on the cover slides were fixed using 4% paraformaldehyde and treated with 0.25% Triton X-100. Subsequent to blocking with 5% normal goat serum for 20 min at 37°C, cells were incubated with rabbit anti-human anti-α-SMA and -E-cadherin monoclonal antibodies (dilution, 1:100; Zhongshan Biotech Co., Ltd.) at 4°C overnight. Cells were incubated with goat anti-rabbit IgG (dilution, 1:100; Boster Biological Technology Co., Ltd.) for 20 min at 37°C and stained with DAB (5 mg/ml; Sigma-Aldrich) for 5 min at room temperature. The specimens then underwent three 5 min washes with PBS and were observed using light microscopy.

### ELISA analysis

The four groups of cells were cultured for 72 h with the solution provided in the ELISA kit (R&D Systems Inc., Minneapolis, MN, USA) according to the manufacturer’s instructions. The absorbance value of each well was read on the full spectrum of the spectrophotometer at a wavelength of 450 nm in order to detect the content of collagen type I (Col I) and fibronectin (FN). Each group consisted of six wells, with the absorbance values averaged over the wells.

### Western blot analysis

The four groups of cells were grown in 10-cm dishes and cultured for 72 h. Cells were then washed with PBS and lysed with lysis buffer (pH 7.4; 1 M Tris-HCl, 1% Triton X-100, sodium deoxycholate and 10% SDS). Solubilized proteins were centrifuged at 14,000 × g at 4°C for 30 min. Extracted proteins were quantified using the Coomassie Protein Assay reagent (Sigma-Aldrich). Western blot analysis of α-SMA and E-cadherin was performed. In brief, 30 μg isolated protein was electrophoresed on 8% sodium dodecyl sulfate-polyacrylamide gels, then transferred (100 V for 1.5 h) onto polyvinylidene fluoride membranes (Gibco-BRL). Membranes were treated with blocking solution [Tris-buffered saline (TBS), pH 7.2; 0.1% Tween-20 and 5% milk] for 1 h, prior to incubation with rabbit anti-human α-SMA, transforming growth factor (TGF)-β1, insulin-like growth factor (IGF)-I, Col I and FN monoclonal primary antibodies (Invitrogen Life Technologies, Carlsbad, CA, USA), diluted 1:1,000 in TBS (pH 7.2) containing 0.1% Tween-20, for 12 h at 4°C. Subsequent to four 15-min washes with TBS (pH 7.2) containing 0.1% Tween-20, the membranes were incubated with the goat anti-rabbit IgG horseradish peroxidase-conjugated antibody (Invitrogen Life Technologies) diluted 1:1,000 in TBS (pH 7.2) with 0.1% Tween-20 for 1 h. Membranes then underwent four 15-min washes with TBS (pH 7.2) and enhanced chemiluminescence (ECL; Gibco-BRL) was performed in accordance with the manufacturer’s instructions. Membranes were then exposed to Kodak X-ray film (R&D Systems Inc.). for 0.5–20 min as required to detect the immunoreactive bands. The relative intensity of the immunoreactive bands on the exposed films was quantified using a computer-assisted densitometry program (SmartView, Major Science, Saratoga, CA, USA). Protein expression was quantified relative to the internal control, glyceraldehyde 3-phosphate dehydrogenase (GAPDH).

### RNA extraction and reverse transcription

Total RNA was extracted from the four groups of cells. In brief, the cells were cultured for 72 h with TRIzol^®^ Reagent (Invitrogen Life Technologies) according to the manufacturer’s instructions and the RNA was dissolved in RNase-free water. The integrity of the RNA was assessed using ethidium bromide agarose gel electrophoresis and the quantity of RNA was determined by measuring the relative absorbance at 260 vs. 280 nm. Complementary (c)DNA was synthesized in a reaction volume of 10 μl using a cDNA synthesis kit (Takara Bio, Inc., Shiga, Japan) according to the manufacturer’s instructions. The cDNA was stored at −20°C.

### Primer preparation

For quantitative polymerase chain reaction (qPCR) amplification, primers (Invitrogen Life Technologies) were derived from the GenBank database. GAPDH was used as the housekeeping gene. The primer sequences were as follows: Forward: 5′-GATGGGCATCTATCA GATAC-3′ and reverse: 5′-AAGCATTTCTGATGGTGATG-3′ for α-SMA; forward: 5′-ATAGAGAACGCATTGC CACATACA-3′ and reverse: 5′-TTCTGATCGGTTACCGTG ATCA-3′ for E-cadherin; forward: 5′-AGGGCCAAGACGAAG ACATC-3′ and reverse: 5′-GTCGGT GGGTGACTCTGAGC-3′ for Col I; forward: 5′-TGGAGGAAGCCGAGGTTT-3′ and reverse: 5′-CAGCGGTTTGCGATGGTA-3′ for FN; and forward: 5′-TGCACCACCAACTGCTTAGC-3′ and reverse: 5′-GGCATGGACTGTGGTCATGAG-3′ for GAPDH.

### qPCR analysis

qPCR reactions were performed using a Brilliant SYBR^®^ Green qRT-PCR Master mix according to the manufacturer’s instructions (Invitrogen Life Technologies). α-SMA, Col I, E-cadherin and FN RNA was amplified using the ABI Prism 7500^®^ Real-Time PCR system (Applied Biosystems, Foster City, CA, USA). qPCR analysis was performed under the following reaction conditions: 95°C for 10 min followed by 40 cycles of 95°C for 15 sec, 60°C for 30 sec, and 72°C for 32 min. The amplified products were subjected to a stepwise increase in temperature from 60°C to 95°C and dissociation curves were constructed.

Target mRNA was quantified by measuring the threshold cycle and reading against a calibration curve. The relative quantity of the target gene mRNA was normalized to that of the housekeeping gene, GAPDH. Results were analyzed using the relative standard curve method of analysis/ΔCt method of analysis.

### Statistical analysis

Data are presented as bar graphs with the mean ± standard deviation of six independent experiments with samples from independent subjects. Data were analyzed using SPSS 15.0 (SPSS, Inc., Chicago, IL, USA) for Windows. Statistical analysis was performed using one-way analysis of variance. P<0.05 was considered to indicate a statistically significant difference.

## Results

### Morphological observation of EECs

**Figure 1:**
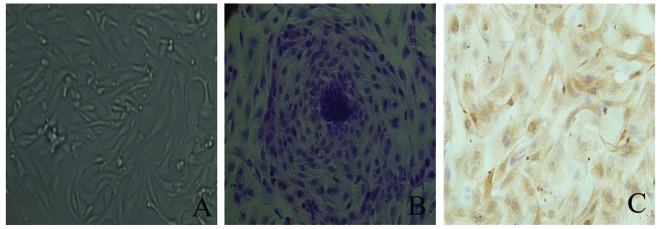
Observation and identification of EECs (magnification, ×200). (A) EECs cultured for five days observed using an inverted phase-contrast microscope. The majority of the EECs formed tightly packed whorls and grew to confluence. (B) EECs stained with hematoxylin and eosin after five days of culture observed using a light microscope. (C) EECs following immunocytochemical staining observed using a light microscope. The EECs were positively stained for cytokeratin using diaminobenzidine. EEC, endometrial epithelium cell.

The EECs were observed using an inverted phase-contrast microscope. The isolated EECs exhibited a round-shaped morphology. The differential centrifugation method revealed that the purity of the EECs was ~90%. Trypan blue staining showed that the viability of the EECs was 92–96%. The cells were found to attach and grow after 24 h of culture and the majority of the EECs formed tightly packed whorls and grew to confluence after 3 days of culture. The EECs exhibited round or elliptical morphology with single large, round nuclei in the center of each cell with a nucleolus (Fig. 1A). The EECs were stained using H&E after 5 days of culture (Fig. 1B).

### Identification of EECs

EECs were detected using immunocytochemical staining. The cytoplasm of EECs, which were positively stained for cytokeratin and negatively stained for vimentin, was stained claybank (Fig. 1C).

### Ang-(1–7) attenuates Ang II-stimulated cell proliferation

**Figure 2:**
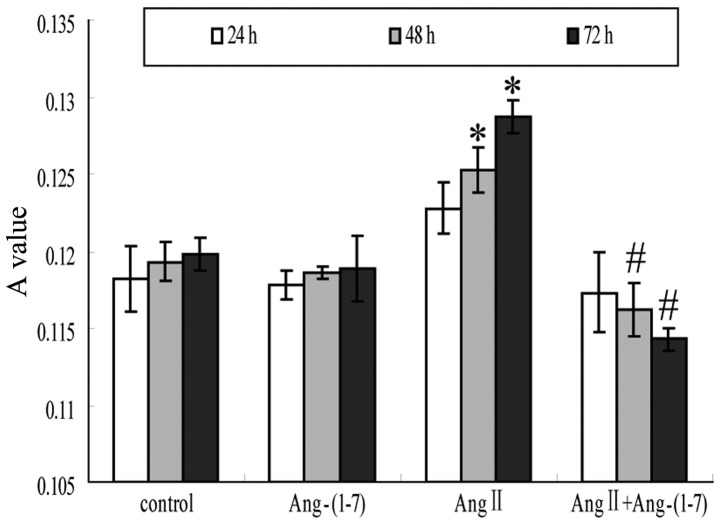
EEC proliferation assessed using an MTT assay. EECs were incubated with different intervention factors for the indicated durations (48–72 h). The groups were as follows: Control, EECs incubated with serum-free DMEM/F12; AngII, EECs incubated with 10^−6^ mol/l AngII and serum-free DMEM/F12; Ang-(1–7), EECs incubated with 10^−5^ mol/l Ang-(1–7) and serum-free DMEM/F12; AngII+Ang-(1–7), EECs incubated with 10^−6^ mol/l AngII and 10^−5^ mol/l Ang-(1–7) and serum-free DMEM/F12. Values are presented as the mean ± standard deviation (n=6). ^*^P<0.05 vs. the control group and ^#^P<0.05 vs. the AngII group. EEC, endometrial epithelium cell; DMEM/F12, Dulbecco’s modified Eagle’s medium/Nutrient Mixture F-12; Ang, angiotensin; A value, absorbance value.

The four groups of EECs were cultured for 24, 48 and 72 h, and the number of EECs in each well were observed by measuring the the absorbance value (A) at 550 nm of the purple crystals in each well. Compared with the control group, the number of EECs in the Ang-(1–7) group was not identified to be significantly different (P>0.05), while the number of EECs in the Ang II group was significantly increased in a time-dependent manner compared with that in the control group (P<0.05). Furthermore, the number of EECs in the Ang II+Ang-(1–7) was significantly reduced compared with that in the Ang II group, suggesting that Ang-(1–7) significantly inhibits Ang II-induced EEC proliferation (Fig. 2).

### Effect of Ang II and Ang-(1–7) on α-SMA and E-cadherin expression in EECs detected using immunocytochemistry

**Figure 3:**
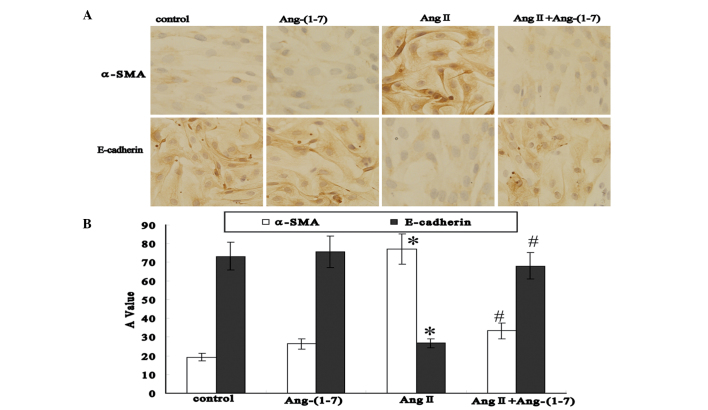
Protein expression of α-SMA and E-cadherin in EECs. EECs were incubated with different intervention factors for 72 h. (A) Immunocytochemistry staining showed brown-yellow granules in the cytoplasm of the EECs (magnification, ×400). (B) Levels of α-SMA and E-cadherin in the EECs, quantified by calculating the average integral A values. The groups were as follows: Control, EECs incubated with serum-free DMEM/F12; AngII, EECs incubated with 10^−6^ mol/l Ang II and serum-free DMEM/F12; Ang-(1–7), EECs incubated with 10^−5^ mol/l Ang-(1–7) and serum-free DMEM/F12; Ang II+Ang-(1–7), EECs incubated with 10^−6^ mol/l Ang II and 10^−5^ mol/l Ang-(1–7) and serum-free DMEM/F12. Values are presented as the mean ± standard deviation (n=6). ^*^P<0.05 vs. the control group and ^#^P<0.05 vs. the Ang II group. α-SMA, α-smooth muscle; EEC, endometrial epithelium cell; Ang, angiotensin; A value; absorbance value.

After the EECs had been cultured for 72 h, immunocytochemistry revealed that the EECs in the control group exhibited low positive expression for α-SMA and high positive expression for E-cadherin. In the Ang-(1–7) group, α-SMA and E-cadherin expression was similar to that in the control group (P>0.05). However, α-SMA expression was observed to be significantly increased in the Ang II group compared with the control group (P<0.05) and significantly decreased in the Ang II+Ang-(1–7) group compared with the Ang II group (P<0.05). Furthermore, E-cadherin expression was found to be significantly decreased in the Ang II group compared with the control group (P<0.05), but significantly increased in the Ang II+Ang-(1–7) group compared with the Ang II group (P<0.05) (Fig. 3).

### Effect of Ang II and Ang-(1–7) on α-SMA and E-cadherin expression in EECs detected using western blot analysis

**Figure 4:**
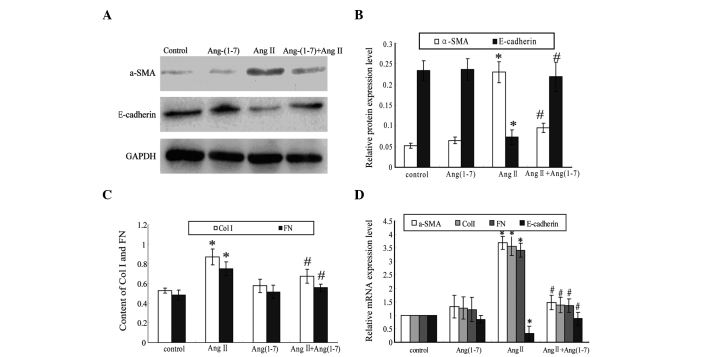
Ang-(1–7) inhibits Ang II-induced EEC transdifferentiation. EECs were incubated with different intervention factors for 72 h. (A) The protein expression of α-SMA and E-cadherin was analyzed using western blot analysis. (B) The protein expression of α-SMA and E-cadherin were quantified using densitometry. (C) The levels of Col I and FN protein in the EEC culture supernatant detected using ELISA. (D) Col I, FN, α-SMA and E-cadherin mRNA expression analyzed using quantitative polymerase chain reaction. The groups were as follows: Control, EECs incubated with serum-free DMEM/F12; Ang II, EECs incubated with 10-6 mol/l Ang II and serum-free DMEM/F12; Ang-(1–7), EECs incubated with 10-5 mol/l Ang-(1–7) and serum-free DMEM/F12; Ang II+Ang-(1–7), EECs incubated with 10-6 mol/l Ang II and 10^−5^ mol/l Ang-(1–7) and serum-free DMEM/F12. Values are presented as the mean ± standard deviation (n=6). ^*^P<0.05 vs. the control group and ^#^P<0.05 vs. the Ang II group. α-SMA, α-smooth muscle; EEC, endometrial epithelium cell; Ang, angiotensin; col I, collagen type I; FN, fibronectin.

After the EECs had been cultured for 72 h, western blot analysis revealed that the EECs in the control group exhibited low α-SMA expression and high E-cadherin expression. α-SMA and E-cadherin expression in the Ang-(1–7) group was found to be similar to that in the control group (P>0.05). However, α-SMA expression was observed to be increased significantly increased in the Ang II group compared with the control group (P<0.05), while significantly decreased in the Ang II+Ang-(1–7) group compared with that in the Ang II group (P<0.05). Furthermore, E-cadherin expression was found to be significantly decreased in the Ang II group compared with the control group (P<0.05), but significantly increased in the Ang II+Ang-(1–7) group compared with that in the Ang II group (P<0.05) (Fig. 4A and B).

### Effect of Ang II and Ang-(1–7) on Col I and FN expression in cultured EEC supernatants detected using ELISA

After the EECs had been cultured for 72 h, ELISA revealed that Col I and FN levels in the EEC culture supernatant in the Ang-(1–7) group showed no significant difference compared with that in the control group (P>0.05). Furthermore, the levels of Col I and FN in the EEC culture supernatant were observed to be significantly increased in the Ang II group compared with the control group (P<0.05), while significantly decreased in the Ang II+Ang-(1–7) group compared with the Ang II group (P<0.05) (Fig. 4C).

### Effect of Ang II and Ang-(1–7) on α-SMA, E-cadherin, Col I and FN expression in EECs detected using qPCR analysis

After the EECs had been cultured for 72 h, qPCR analysis revealed that compared with those of the control group, the mRNA levels of α-SMA, E-cadherin, Col I and FN in the Ang-(1–7) group showed no significant change. However, compared with the control group, in the Ang II group, α-SMA, Col I and FN expression were found to be significantly increased and E-cadherin expression was observed to be significantly decreased (P<0.05). Furthermore, compared with the Ang II group, in the Ang II+Ang-(1–7) group, α-SMA, Col I and FN expression were significantly decreased, while E-cadherin expression was significantly increased (P<0.05) (Fig. 4D).

## Discussion

IUAs are caused by numerous factors and are characterized by the proliferation of ESCs and the excessive accumulation of extracellular matrix components. Mechanisms involved in the development of IUAs include: (i) Inflammatory, immune and toxic stimuli-induced ESC and EEC activation, causing proliferation and transdifferentiation into myofibroblast cells; (ii) increases in extracellular matrix components and decreases in extracellular matrix degradation; and (iii) the involvement of certain vascular active substances, including Ang II, as well as TGF-β1 and cytokines. The disappearance of a large number of EECs during the development of IUA, may be associated with EEC transdifferentiation into myofibroblasts.

EEC transdifferentiation is important during the development and progression of IUA. During EEC transdifferentiation under certain pathological conditions, EECs lose the expression of epithelial markers, including cytokeratin and E-cadherin, and begin to express mesenchymal cell markers, including α-SMA and vimentin. The transformation of the cell phenotype often loses control and leads to excessive proliferation or hypertrophy. Furthermore, secretion and degradation of extracellular matrix components often loses balance and excessive secretion of cytokines, inflammatory chemokines or cell adhesion molecules occurs. These events are directly involved in the process of endometrial fibrosis.

As EECs and stromal cells originate from the same embryonic stem cell, under certain pathological conditions, the transdifferentiation of EECs to mesenchymal cells can occur, with EECs transdifferentiating into myofibroblasts with increased α-SMA expression and migrating through the basement membrane into the lamina propria (16). Myofibroblasts have a role in tissue fibrosis and have a higher capacity for proliferation and the secretion of ECM components compared with ordinary fibroblasts. Myofibroblasts initially secrete FN, which provides support for the sedimentation of other ECM components and the formation of collagen fibers. Myofibroblasts then secrete collagen, primarily type I and type III collagen, as well as laminin and proteoglycans. Thus, ECM generation is greater than ECM degradation, eventually leading to endometrial fibrosis.

Ang II not only promotes the proliferation of various types of mesenchymal cell, but also causes the accumulation of extracellular matrix components, leading to fibrosis. A study demonstrated that Ang II is capable of inducing cardiomyocyte hypertrophy and myocardial interstitial fibrosis (5). Ang II has also been reported to stimulate renal tubular epithelial cells to produce TGF-β and to promote the expression of fibrotic factors, including connective tissue growth factor, basic fibroblast growth factor, PAl-1, as well as promote the expression of platelet-derived growth factor (PDGF), which aggravate renal interstitial fibrosis (17). The present study showed that *in vitro*, Ang II promotes proliferation and activation of EECs, and significantly increases the expression of α-SMA protein and α-SMA mRNA (P<0.05), while Ang II significantly decreases the expression of E-cadherin protein and mRNA (P<0.05). These findings suggest that Ang II promotes cells with EEC phenotypes to transdifferentiate into cells with myofibroblast phenotypes. Furthermore, in the present study, Ang II was observed to significantly increase the expression of Col I and FN mRNA in EECs and EEC culture supernatant (P<0.05), suggesting that Ang II promotes the secretion of extracellular matrix proteins, including FN and Col I, consistent with the results. Therefore, Ang II may have an important role in promoting endometrial fibrosis.

Ang-(1–7) is a novel member of the RAAS and is an endogenous antagonist of Ang II, which may exhibit an anti-fibrotic effect by inhibiting cell proliferation and extracellular matrix accumulation. Le Tran and Forster (18) were the first to identify that Ang-(1–7) is capable of inhibiting the effect of Ang II, FBS and PDGF on the proliferation of smooth muscle cells. Tallant *et al* (14) also showed that Ang-(1–7) inhibits the effect of FBS and endothelin-1 on the proliferation of newborn rat cardiac fibroblasts and on myocyte hypertrophy. Furthermore, Zhang *et al* (19) identified that Ang-(1–7) inhibits the effect of Ang II on the proliferation of vascular smooth muscle cells. The rat model of carotid artery injury was treated with Ang (1–7) (24 pg/kg/h) for 12 days, and the results demonstrated that Ang (1–7) can reduce the neointimal area and significantly reduce DNA synthesis of membrane cells (mainly in smooth muscle cells). These findings show that Ang-(1–7) is capable of inhibiting the proliferation of smooth muscle cells *in vivo* and may prevent restenosis following angioplasty (20). Ang-(1–7) has also been shown to inhibit the effect of Ang II on fibrosis of skeletal muscle cells, as well as reduce the expression of TGF-β1 induced by Ang-II in a dose-dependent manner (21). TGF-β1 is one of the predominant cytokines involved in causing fibrosis, thus the inhibitory effect of Ang-(1–7) on TGF-β1 inhibition may be an important anti-fibrotic mechanism in the body. Burns *et al* (7) further showed that Ang-(1–7) inhibits Ang II-induced transdifferentiation of renal tubular epithelial cells and the expression of extracellular matrix proteins, using the joint intervention of Ang II and Ang-(1–7) in rat renal tubular epithelial cells. The present study identified that *in vitro*, Ang-(1–7) had an inhibitory effect on Ang II-induced EEC proliferation and Ang-(1–7) inhibited Ang II-induced α-SMA expression (P<0.05). Furthermore, Ang-(1–7) was observed to inhibit the Ang II-induced decrease in E-cadherin mRNA and protein expression (P<0.05). These findings suggest that Ang-(1–7) is capable of inhibiting Ang II-induced EEC transdifferentiation at the protein and gene level. Compared with the Ang II group, the expression of Col I and FN mRNA and the levels of FN and Col I in the EEC culture supernatant were found to decrease significantly in the Ang-(1–7)+Ang II group (P<0.05). This indicates that Ang-(1–7) inhibits the Ang II-induced secretion of FN and Col I in EECs, suggesting Ang-(1–7) is capable of reducing the synthesis of ECM components, inhibiting the occurrence and development of endometrial fibrosis, thus delaying the process of endometrial fibrosis.

In conclusion, Ang-(1–7) is capable of inhibiting Ang II-induced transdifferentiation of EECs into myofibroblasts and Ang-(1–7) inhibits the Ang II-induced secretion of extracellular matrix components, including FN and Col I. This has important significance for the prevention and treatment of endometrial fibrosis in the clinic.
